# Protein Tyrosine Phosphatase SHP2 Controls Interleukin-8 Expression in Breast Cancer Cells

**DOI:** 10.1007/s10911-022-09521-x

**Published:** 2022-06-23

**Authors:** Romain J. Amante, Priska Auf der Maur, Veronica Richina, Atul Sethi, Vytautas Iesmantavicius, Debora Bonenfant, Nicola Aceto, Mohamed Bentires-Alj

**Affiliations:** 1 Department of Biomedicine, University of Basel, University Hospital Basel, Basel, Switzerland; 2 Friedrich Miescher Institute for Biomedical Research, Basel, Switzerland; 3 Analytical Sciences and Imaging, Novartis Institutes for Biomedical Research, Basel, Switzerland; 4 Department of Biology, Institute of Molecular Health Sciences, Swiss Federal Institute of Technology (ETH) Zurich, Zurich, Switzerland

**Keywords:** Breast cancer, Cytokines, CXCL8, ETS1, IL-8, MAPK, Microenvironment, Phosphatase, PTPN11, SHP2

## Abstract

Treatment of metastasis remains a clinical challenge and the majority of breast cancer-related deaths are the result of drug-resistant metastases. The protein tyrosine phosphatase SHP2 encoded by the proto-oncogene *PTPN11* promotes breast cancer progression. Inhibition of SHP2 has been shown to decrease metastases formation in various breast cancer models, but specific downstream effectors of SHP2 remain poorly characterized. Certain cytokines in the metastatic cascade facilitate local invasion and promote metastatic colonization. In this study, we investigated cytokines affected by SHP2 that could be relevant for its pro-tumorigenic properties. We used a cytokine array to investigate differentially released cytokines in the supernatant of SHP2 inhibitor-treated breast cancer cells. Expression of *CXCL8* transcripts and protein abundance were assessed in human breast cancer cell lines in which we blocked SHP2 using shRNA constructs or an allosteric inhibitor. The impact of SHP2 inhibition on the phospho-tyrosine-proteome and signaling was determined using mass spectrometry. From previously published RNAseq data (Aceto et al. in Nat. Med. 18:529–37, 2012), we computed transcription factor activities using an integrated system for motif activity response analysis (ISMARA) (Balwierz et al. in Genome Res. 24:869–84, 2014). Finally, using siRNA against *ETS1*, we investigated whether ETS1 directly influences *CXCL8* expression levels. We found that IL-8 is one of the most downregulated cytokines in cell supernatants upon SHP2 blockade, with a twofold decrease in *CXCL8* transcripts and a fourfold decrease in IL-8 protein. These effects were also observed in preclinical tumor models. Analysis of the phospho-tyrosine-proteome revealed that several effectors of the mitogen-activated protein kinase (MAPK) pathway are downregulated upon SHP2 inhibition in vitro. MEK1/2 inhibition consistently reduced IL-8 levels in breast cancer cell supernatants. Computational analysis of RNAseq data from *SHP2*-depleted tumors revealed reduced activity of the transcription factor ETS1, a direct target of ERK and a transcription factor reported to regulate IL-8 expression. Our work reveals that SHP2 mediates breast cancer progression by enhancing the production and secretion of the pro-metastatic cytokine IL-8. We also provide mechanistic insights into the effects of SHP2 inhibition and its downstream repercussions. Overall, these results support a rationale for targeting SHP2 in breast cancer.

**Supplementary Information:**

The online version contains supplementary material available at 10.1007/s10911-022-09521-x.

## Background

Breast cancer accounts for most cancer-related deaths among women worldwide [3]. A third of all breast cancers progress to metastasis, which remains the major cause of death in patients with solid tumors [4, 5]. Elucidation of the molecular mechanisms of tumor progression is essential in order to design treatment strategies. Blockade of the tyrosine phosphatase Src-homology 2 domain-containing phosphatase (SHP2) has been shown to reduce metastatic spread [1, 6] in animal models. SHP2 is a signal-enhancing phosphatase downstream of several receptor tyrosine kinases (such as epidermal-, hepatocyte- and fibroblast-growth factor receptors) [7]. We asked whether and how SHP2 regulates cytokines that could be relevant for its pro-tumorigenic properties in breast cancer.

Tumor heterogeneity on intra- and inter patient level remains a major challenge for treatments success [8, 9]. Such heterogeneity exists on genetic and phenotypic levels. Intratumor heterogeneity yields cells with several molecular and cellular programs that ultimately increase the likelihood of aggressive clones with enhanced survival, invasion and colonization [10]. Cancer cells secrete a plethora of cytokines and chemokines, often at excessive levels compared to physiological values. Those secreted factors can vary greatly between different breast cancer subtypes. Release of these factors may modulate the surrounding stroma towards a pro-tumorigenic microenvironment [11]. The CXC chemokine IL-8 belongs to the CXC glutamic acid-leucine-arginine motif-bearing family and binds to its cognate receptors CXCR1 and CXCR2 [12]. IL-8 promotes inflammation and tumor initiation and is involved in metastasis [13–15]. In addition, we have previously demonstrated that IL-8 is an active factor in breast cancer cell invasion especially in TNBC and HER2^+^ breast cancer models [16, 17]. Furthermore, IL-8 has been reported to increase cancer cell proliferation, survival, and resistance to chemotherapy, as well as enhancing angiogenesis and immune-cell recruitment [15].

The evolutionarily conserved family of E26 transformation-specific (ETS) transcription factors regulate fundamental cellular processes such as proliferation, differentiation, apoptosis, and migration [18]. In cancer, frequent dysregulation of this family leads to aberrant gene-expression programs that contribute to tumorigenesis [16, 18]. Particularly, ETS proto-oncogene 1 (*ETS1*) is a known oncogenic transcription factor overexpressed in breast cancer [19] that is driven by the mitogen-activated protein kinase (MAPK) [20].

We investigated cytokines affected by SHP2 to determine whether they contribute to its pro-tumorigenic effects. By interfering with the SHP2/MAPK axis and applying global omics approaches, our study brings new insights into how SHP2 influences IL-8 secretion in different breast cancer cell lines and thereby promotes breast cancer progression. We found that IL-8 expression is enhanced by SHP2 via the MAPK pathway and the ETS1 transcription factor. These results support previous efforts to target SHP2 in breast cancer to reduce cancer progression and ultimately prolong survival [1].

## Results

### Inhibition of SHP2 Downregulates Expression of IL-8 in Breast Cancer Cell Lines

**Fig. 1:**
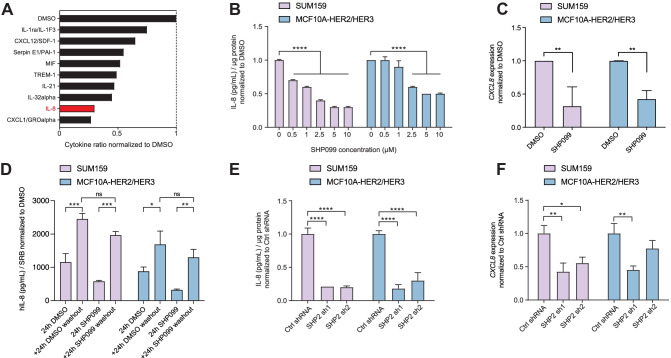
SHP2 blockade downregulates IL-8. (**A**) Bar graph representing the quantification of the nine most downregulated cytokines in SUM159 cell supernatants treated with SHP099 for 48 h relative to vehicle-treated cells. Data shown are dot quantification by pixel density from cytokine-array scans. (**B**) Bar graph representing IL-8 protein abundance in the supernatant of the indicated cell lines treated for 48 h at the SHP099 concentrations shown. Data are IL-8 protein concentrations normalized by total protein ± standard deviation (S.D.) (*n* = 3, ****P ≤ 0.0001, Two-way ANOVA test). (**C**) Bar graph representing *CXCL8* mRNA expression in the indicated cell lines after 120 h of SHP099 inhibition (*n* = 3, **P ≤ 0.001, Two-way ANOVA test). (**D**) Bar graph representing IL-8 protein abundance in the supernatant of the indicated cell lines treated for 24 h with DMSO or 10 µM SHP099 normalized to the protein content of each well determined by SRB staining. Washout conditions were collected 24 h after media exchange. (*n* = 3, *P ≤ 0.05, **P ≤ 0.01, ***P ≤ 0.001, Two-way ANOVA test). (**E**) Bar graph representing IL-8 protein abundance in the supernatants of the indicated cell lines upon *SHP2* knockdown. Data are shown ± S.D. (*n* = 3, ****P ≤ 0.0001, Two-way ANOVA test). (**F**) Bar graph representing *CXCL8* mRNA expression in the indicated cell lines upon *SHP2* knockdown. Data are shown ± S.D. (*n* = 3, *P ≤ 0.05, **P ≤ 0.01; Two-way ANOVA test)

We first investigated the effect of the allosteric SHP2 inhibitor SHP099 on the secreted chemokines of the triple-negative breast cancer (TNBC) cell line SUM159. Cytokine array analysis revealed downregulation of several cytokines in conditioned medium from SUM159 treated with SHP099, including CXCL1, IL-8, and IL-32α, with decreases of 73%, 70%, and 55%, respectively (Fig. 1A).

We then performed IL-8 ELISA on supernatants from SUM159 and MCF10A-HER2/HER3 cells treated with SHP099 or vehicle control at different concentrations. In the SUM159 cells, the IL-8 level was 30% lower than the control at 0.5 µM and 70% lower at 5 µM. In contrast, in MCF10A-HER2/HER3 cells, IL-8 expression was lower by 40% at 2.5 µM and 50% at 5 µM (Fig. 1B).

To investigate whether SHP2 affects *CXCL8* at the transcriptional level, we measured *CXCL8* transcripts by real-time quantitative PCR in SUM159 and MCF10-HER2/HER3 treated with SHP099. After 120 h of SHP2 inhibition, *CXCL8* mRNA levels were lower in SUM159 and MCF10A-HER2/HER3 cells by 68% and 57%, respectively (Fig. 1C).

To explore cell recovery after SHP2 inhibition using SHP099, we have performed a washout experiment. We replaced the medium containing DMSO or SHP099 after 24 h, with fresh medium (without DMSO or SHP099) for the SUM159 and MCF10A-HER2/HER3 cell lines (Fig. 1D). Both cell lines showed a significant IL-8 increase after fresh medium exchange, regardless of conditions (DMSO or SHP099).

Furthermore, short hairpin-mediated depletion of *SHP2* (SHP2 sh) (Supplementary Fig. 1A) also reduced both the expression and protein abundance of IL-8 when SUM159 and the MCF10A-HER2/HER3 cells were grown as a monolayer (Fig. 1E, F) or as 3D cultures (Supplementary Fig. 1B). Thus, knockdown or allosteric inhibition of SHP2 reduces IL-8 expression and secretion.

### SHP2 Blockade Lowers IL-8 Levels by Inhibiting the MAPK Signaling Cascade and Leads to Reduced ETS1 Activity

**Fig. 2:**
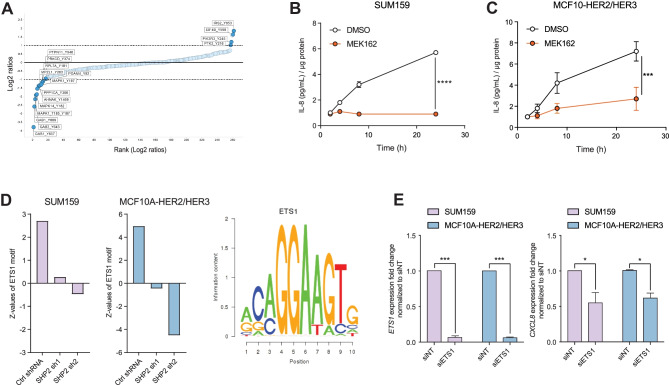
SHP2/MAPK enhances IL-8 levels via the transcription factor ETS1. (**A**) Scatter plot representing ranked phospho-tyrosine peptides depleted (left) or enriched (right) in SUM159 cells after 30 min of SHP099 treatment. Dark blue dots indicate phospho-peptides known to be involved in MAPK pathway regulation. The dotted line represents an arbitrary cutoff value. (**B**–**C**) Curves representing IL-8 protein abundance in the supernatants of the indicated cell lines upon MEK162 treatment at the indicated time points. Data shown are IL-8 protein concentrations normalized by total protein. Fold changed based on the 2h time point, ± S.D. (*n* = 3, ***P ≤ 0.001, ****P ≤ 0.0001, Two-way ANOVA test). (**D**) Bar graphs showing the ETS1 transcription factor activity profile (left) in *SHP2* knockdown SUM159 tumors and MCF10A-HER2/HER3 3D cultures and its DNA-binding motif (right). (**E**) Bar graphs representing *ETS1* and *CXCL8* mRNA expression in the indicated cell lines 96 h after addition of siRNA (*n* = 3, *P ≤ 0.05, ***P ≤ 0.001, Two-way ANOVA test)

To investigate how SHP2 activity reduces IL-8 levels, we performed short SHP099 treatments from 1 to 30 min in SUM159 cells and quantified phospho-tyrosine peptides by mass spectrometry (Fig. 2A; Supplementary Fig. 1C). Of the significantly impacted phospho-tyrosine peptides, 17 are involved in the MAPK pathway regulation. Of these, 13 peptides were less abundant and 4 peptides were more abundant than in vehicle-treated SUM159 (Fig. 2A; Supplementary Fig. 1D). Separate analysis showed that the phospho-serine and phospho-threonine peptides downregulated upon SHP2 inhibition were all peptides of proteins involved in the regulation of the MAPK pathway (Supplementary Fig. 1D). Immunoblotting confirmed that SHP2 inhibition by SHP099 reduced phosphorylation of ERK1 and ERK2 (Thr202/Tyr204, respectively), two downstream effectors of active MAPK signaling (Supplementary Fig. 1F). Together this indicates that SHP099 inhibits MAPK signaling.

SHP2 is required for MAPK activation downstream of most receptor tyrosine kinases [21]. To assess whether MAPK signaling regulates IL-8, we treated SUM159 and MCF10A-HER2/HER3 with the MEK1/2 inhibitor MEK162. MEK inhibition reduced IL-8 concentration in the supernatant 6.3-fold compared to vehicle in SUM159 cells and 2.7-fold in MCF10A-HER2/HER3 cells (Fig. 2B, C). The data suggest that SHP2 inhibition of MAPK signaling may account for the reduction in IL-8 expression and abundance.

To identify the transcription factors that regulate IL-8 expression downstream of SHP2/MAPK, we performed a Motif Activity Response Analysis (MARA) on RNAseq data from SUM159 tumors and MCF10A-HER2/HER3 cells grown in 3D cultures in the presence or absence of SHP2. The MARA results indicated that the activity of the transcription factor ETS1 was reduced by *SHP2* knockdown (Fig. 2D).

To assess whether ETS1 directly modulates IL-8 expression, we transfected SUM159 and MCF10A-HER2/HER3 cells with small interfering RNA (siRNA) targeting *ETS1* and quantified *CXCL8* transcripts by RT-qPCR. Knockdown of *ETS1* in both models reduced expression of *CXCL8* (Fig. 2E).

## Discussion

Cancer cells frequently show enhanced chemokine signaling, which in turn supports their proliferation and survival [15]. In this study, we investigated cytokines regulated by SHP2 that may further contribute to pro-tumorigenic properties. We found decreases in *CXCL8* transcripts and protein after *SHP2* knockdown or after pharmacological inhibition. MEK1/2 inhibition-evoked decrease in MAPK signaling also reduced levels of the cytokine IL-8. Thus, the fact that SHP2 blockade reduces MAPK activity suggests this as a mechanism through which it dampens IL-8 levels.

Computational analysis of RNAseq data from tumors following knockdown of *SHP2* revealed reduced activity of the transcription factor ETS1, a direct target of ERK and a reported transcription factor of *CXCL8* [22–25] (see Supplementary Fig. 1G). Knockdown of *ETS1* indeed reduced the levels of *CXCL8*, which suggests that it is regulated via the SHP2/MAPK/ETS1. Several other transcription factors (AP1, NF-kB) are also known to regulate IL-8 expression in a cell type-specific manner [26, 27], which may account for the levels of IL-8 still detected after SHP2 inhibition.

Under physiological conditions, IL-8 is secreted by myeloid cells, endothelial cells, and fibroblasts. It recruits granulocytes and contributes to the resolution of infections and the healing of damaged tissue [28]. IL-8 has a broader effect in tumors. In a paracrine manner, the IL-8/CXCR1/CXCR2 axis attracts neutrophils and myeloid-derived suppressor cells (MDSC), resulting in an immunosuppressive and pro-tumorigenic environment. These immune infiltrates secrete growth factors and cytokines, remodel the extracellular matrix and induce angiogenesis [29, 30]. In an autocrine fashion, tumor-derived IL-8 facilitates oncogenic signaling, angiogenesis, epithelial-to-mesenchymal transition (EMT) [31], acquisition of stem-cell traits, resistance to therapy, and pro-metastatic features of cancer cells [13, 14, 17, 32]. Also, the microenvironment has a dramatic impact on cancer cells and their exposure to IL-8 [33]. Cancer cells in the periphery of the tumor bulk are in closer contact with fibroblasts, hence exposed to IL-8 and its effects [34]. This adds an extra layer of complexity leading to tumor heterogeneity [9].

In clinical settings, IL-8 is upregulated in numerous human tumors (e.g. brain, breast, lung, melanoma) [35]. High levels of this cytokine in the serum of cancer patients often correlate with an adverse prognosis [29, 36] and predict a poor response to checkpoint-based immunotherapy [37]. For example, in colon cancer mouse models, the combination of an SHP2 inhibitor and an anti-PD-1 antibody showed higher therapeutic efficacy than monotherapy [38]. It remains to be determined whether this is mediated by the effect of SHP2 inhibition on IL-8 levels and whether a similar combination would be beneficial in other cancer types.

Our cytokine array showed that other cytokines such as CXCL1 and IL32-alpha are downregulated upon SHP2 inhibition in breast cancer cell lines. CXCL1 also mediates neutrophil recruitment and activation [39] and, similar to IL-8, was reported to support cancer growth, angiogenesis, and metastasis [40]. IL-32 is a pro-inflammatory cytokine involved in cancer-related inflammation and is upregulated in several malignancies [41]. The contribution of these cytokines to the anticancer effects of SHP2 inhibition is warranted.

We and others have previously demonstrated a fundamental effect of SHP2 on tumor maintenance and progression in HER2-positive and triple-negative breast cancers [6, 7, 16, 42]. Given that both IL-8 and ETS1 expression correlate with the invasive phenotypes of cancer cells and angiogenesis [19, 43], our study provides further evidence of the multifaceted promotion of breast cancer progression by SHP2, in this case via upregulation of the pro-tumorigenic cytokine IL-8.

## Methods

### Cells, Cell Culture, and Reagents

SUM159 cells were propagated in Nutrient Mixture F-12 supplemented with 5% fetal calf serum, 0.5 μg/ml hydrocortisone, and 10 μg/ml insulin (all from Sigma), 100 IU/ml penicillin, 100 μg/ml streptomycin, and 100 μg/ml Normocin (InvivoGen). MCF10A-HER2/HER3 [1] were propagated in DMEM/F12 medium (Invitrogen) supplemented with 5% horse serum (Hyclone), 20 ng/ml human EGF (Peprotech), 0.5 μg/ml hydrocortisone, 100 ng/ml cholera toxin, and 10 μg/ml insulin (all from Sigma), 100 IU/ml penicillin, 100 μg/ml streptomycin and 100 μg/ml Normocin (InvivoGen). These cells were profiled for cell line-specific highly-polymorphic short tandem repeat loci (STRs) (Microsynth). Previously published tools were used for the inducible RNAi studies [1]. For siRNA experiments, 175,000 cells were seeded in 6-well plates. The following day, cells were transfected using DharmaFECT and the human ETS1 siRNA- SMARTpool (L-003887-00-0005) or the non-targeting pool as control (D-001810-10-05). Experiments were performed according to the manufacturer’s protocol and using siRNA at final concentrations of 25 nM and 12.5 nM for SUM159 and MCF10A-HER2/HER3, respectively. Cells were harvested at the times indicated in the figure legends.

### Compounds

SHP099 and MEK162 were obtained from Novartis (Basel, Switzerland and Cambridge, USA). Compounds were prepared as 10 mM stock solutions in DMSO and stored protected from light at –20 °C.

### Cytokine Array and IL-8 ELISA

Cells were cultured overnight in 6-well plates at 250,000 cells/well and the culture medium containing the inhibitor(s) described above was then added. Cell supernatants were collected 48 h later and cells lysed with RIPA buffer (50 mM Tris-HCl pH 8, 150 mM NaCl, 1% NP-40, 0.5% sodium deoxycholate, 0.1% SDS) supplemented with 1 × protease inhibitor cocktail (Complete Mini, Roche), 0.2 mM sodium orthovanadate, 20 mM sodium fluoride, and 1 mM phenylmethylsulfonyl fluoride (Fig. 1A–C). For washout experiments, cells were seeded in 96-well plate at 10’000 cells/well for MCF10A HER2/HER3 and 5’000 cells/well for SUM159. The day after, culture medium containing DMSO or 10 μM SHP099 was added. After 24 h, cell supernatants were collected, and fresh media was added (washout), and cell supernatant was harvest again after 24 h [44]. For protein quantification, cells were fixed and stained for SRB (Fig. 1D). For *SHP2* knockdown cell lines, a similar time plan was used, following 5 days of doxycycline treatment (Fig. 1E, F). Cytokine arrays were performed using a Proteome Profiler Human Cytokine Array Kit (R&D Systems) according to the manufacturer’s protocol. IL-8 ELISA was performed using the Legend Max Human IL-8 ELISA Kit with Pre-coated Plates (BioLegend) according to the manufacturer’s protocol.

### Immunoblotting

Treated cells were lysed in RIPA buffer (50 mM Tris-HCl pH 8, 150 mM NaCl, 1% NP-40, 0.5% sodium deoxycholate, 0.1% SDS) containing 1 × protease inhibitor cocktail (Complete Mini, Roche), 0.2 mM sodium orthovanadate, 20 mM sodium fluoride, and 1 mM phenylmethylsulfonyl fluoride. Samples were supplemented with Laemmli buffer and boiled for 5 min at 95 °C on a heating block. Proteins (30 µg) were loaded onto a 10% polyacrylamide gel and subsequently transferred to a PVDF membrane (Immobilon-P, Millipore). The membrane was blocked for 1 h at room temperature with 5% BSA in TBS supplemented with 0.05% Tween 20 (TBS-T). Primary antibodies (anti-phospho-p44/42 MAPK (Erk1/2) (Thr202/Tyr204) Cell Signaling, #9101; anti-ERK2 Santa Cruz Biotechnology, sc-1647) were diluted in TBS-T and incubated with the membrane overnight at 4 °C. Secondary antibodies (IRDye 680RD or 800RD) were incubated with the membrane for 1 h at room temperature. Blots were imagined using a LI-COR Odyssey CLx imager.

### Proteomic Analysis

SUM159 cells were cultured in 500-cm^2^ Square TC-treated Culture Dishes (Corning) to 65% confluence. For each sample, three plates have been treated with DMSO for the control sample and with 10 µM of SHP099 at 1 min, 5 min, 15 min and 30 min time points. After rinsing the plates with ice-cold PBS twice, cells were collected into 2% sodium deoxycholate in 50 mM HEPES buffer (pH 8.5) using a cell scraper. The cell lysate was sonicated using a Branson digital tip sonicator for 1 min at 70% amplitude on ice, followed by a 5-min incubation at 95 °C. Proteins were reduced and alkylated for 30 min in the dark in 5 mM TCEP or 5 mM chloroacetamide, respectively. The samples were fourfold diluted with 50 mM HEPES buffer (pH 8.5) and digested overnight with LysC (Wako Chemicals) at a 1:100 ratio. The next morning, samples were supplemented with trypsin (ThermoFisher) at a 1:100 ratio and incubated for 24 h at 37 °C. The digestion was quenched by adding 10% TFA to a final concentration of 1%. The peptides were cleared by centrifugation for 5 min at 7000 × *g*, purified using a SEP-PAK (Waters), and eluted in 50% acetonitrile in water.

For phosphotyrosine enrichment, 6.5 mg aliquots of peptides from each sample were subjected to immunoprecipitation using the PTMScan Phospho-Tyrosine Motif Kit from Cell Signaling (P-Tyr-1000) and the phosphorylated peptides were enriched and eluted according to the manufacturer’s instructions. Eluted peptides were labeled with TMT10plex isobaric labeling reagents (Thermo Fisher) as described in the manufacturer’s instructions, followed by off-line high pH fractionation.

For the global proteome and the phosphoserine/threonine enrichment, a peptide fraction of 500 µg from each sample was labeled with TMT reagents and pooled. A 100 µg aliquot of the peptide mixture was subjected to off-line high pH fractionation for global proteome measurements, while the rest was used for TiO2-based phosphorylated peptide enrichment as described in Borisova et al. [45], followed by off-line high pH fractionation of phosphorylated peptides. The high pH off-line fractionation was carried out on a YMC Triart C18 0.5 × 250 mm column (YMC Europe GmbH) using the Agilent 1100 system (Agilent Technologies). A total of 96 fractions was collected for each experiment and concatenated into 48 fractions as previously described [46]. For each LC-MS analysis, approximately 1 µg aliquots of peptides were loaded onto PepMap 100 C18 2 cm trap (Thermo Fisher) using the Proxeon NanoLC-1000 system (Thermo Fisher). On-line peptide separation was performed on a 15 cm EASY-Spray™ C18 column (ES801, Thermo Fisher) by applying a linear gradient of increasing ACN concentration at a flowrate of 150 nL/min. Orbitrap Fusion Lumos Tribrid (Thermo Fisher) mass spectrometer was operated in the data-dependent mode. The ions for the survey scan were collected for a maximum of 100 ms to reach the AGC target value of 20’0000 and the scan recorded using an Orbitrap detector at a resolution of 120’000. The top 10 most intense precursor ions from the Orbitrap survey scan were selected for higher-energy C-trap dissociation (HCD) at 38% normalized collision energy scan. To reach an AGC value of 50’000 ions, the maximum ion accumulation time for the MS2 scan was set to 180 ms for the proteome measurements and 150 ms for phosphorylated peptide measurements. The TMT reporter ions were quantified using an MS2 scan recorded using the Orbitrap analyzer at a resolution of 50’000. Thermo RAW files were processed using Proteome Discoverer 2.1 software (Thermo Fisher) as described in the manufacturer’s instructions. Briefly, the Sequest search engine was used to search the MS2 spectra against the *Homo sapiens* UniProt database (downloaded on 04/04/2017) supplemented with common contaminating proteins. For total proteome analysis, cysteine carbamidomethylation and TMT tags on lysine and peptide N-termini were set as static modifications, whereas oxidation of methionine residues and acetylation protein N-termini were set as variable modifications. For phosphorylated peptide-enriched sample analysis, serine, threonine, and tyrosine phosphorylation were set as variable modifications while other modifications were set as for the proteome analysis. The assignments of the MS2 scans were filtered to allow 1% FDR. For reporter quantification, the S/N values were corrected for isotopic impurities of the TMT reagent using the values provided by the manufacturer. The sums across all TMT reporter channels were normalized assuming equal total protein content in each sample for proteome analysis whereas, for phosphorylated peptide analysis, normalization was based on the total amount of phosphorylated peptides.

### Microarray Analysis

Total RNA was extracted from SUM159 samples with TRIzol reagent (Invitrogen), processed and hybridized to GeneChip Human Gene 1.0 ST arrays (Affymetrix, Santa Clara, CA), and scanned according to the manufacturer's instructions. CEL files for MCF10a samples were downloaded from Gene Expression Omnibus repository GSE34525 (https://www.ncbi.nlm.nih.gov/geo/query/acc.cgi?acc=GSE34525).

All gene arrays were processed in R (http://www.r-project.org/) using Bioconductor and the package oligo [47]. Robust multi-array mean was performed using the following command: expr <- rma(read.celfiles(filenames)). Probes with the largest interquartile range were selected as representative of corresponding genes (using array annotation from Bioconductor package hugene10sttranscriptcluster.db). Microarray data for SUM159 are accessible from the Gene Expression Omnibus repository (GSE182033). Differential gene expression between cells engineered with Crtl shRNA, SHP2 sh1, and SHP2 sh2 was calculated using the package limma [48].

### Motif Activity Response Analysis (MARA)

We used the MARA [2] to model genome-wide gene expression patterns in terms of computationally predicted transcription factor binding sites. We compared the activity means and standard deviations of several regulatory motifs under control and *SHP2*-knockdown conditions.

### Quantitative Real-Time PCR

Total RNA was extracted using the RNeasy Plus Mini Kit (Qiagen) and reverse transcribed using the iScript cDNA Synthesis Kit (BioRad). The resulting cDNA was used for TaqMan-based quantitative real-time PCR using the PrimeTime Gene Expression Master Mix (IDT) for quantification of *CXCL18*, *PTPN11*, and *ETS1*, with *HPRT1* as a control gene. The following PrimeTime qPCR Probe Assays (IDT) were used: Hs.PT.58.39926886.g, Hs.PT.56a.20552233, Hs.PT.58.39917763, Hs.PT.58v.45621572.

### Statistical Analysis

In each of the studies presented, the results shown represent at least three independent experiments. Values are reported as means ± standard deviation. Data were tested for normal distribution and ANOVAs tests were applied using GraphPad Prism 7.04. The *P* values < 0.05 were considered statistically significant.

### Data Availability

Proteomic data are available via ProteomeXchange with identifier PXD017219. Microarray data were described elsewhere [1] and can be downloaded from the Gene Expression Omnibus repository under GSE34525 and GSE182033 for the MCF10A and SUM159, respectively.

## Supplementary Information

Below is the link to the electronic supplementary material.

Supplementary file 1: SHP2 blockade downregulates MAPK and leads to reduced IL-8 levels in vitroand in vivo*. *(**A**) Bar graph representing *PTPN11* (SHP2) mRNA expression in the indicated cell lines upon SHP2 knockdown. Data are shown ± S.D.(*n *= 3, ****P ≤ 0.0001, Two-way ANOVA test). (**B**) Differential expression of *PTPN11* and *CXCL8* upon SHP2 knockdown in SUM159 tumors and MCF10A-HER2/HER3 3D cultures. (**C**) Scatter plot representing ranked phospho-tyrosine peptides depleted (left) or enriched (right) in SUM159 cells upon SHP099 treatment at the indicated timepoints. Dark blue dots indicate phospho-peptides known to be involved in MAPK pathway regulation. The dotted line represents the used cutoff value of 1-fold change in log2 space. (**D**) Representative phospho-tyrosine peptides involved in MAPK pathway activation (orange) or inhibition (blue) upon SHP099 inhibition at the indicated time points. (**E**) Scatter plot representing phospho-serine/threonine peptides depleted (left) or enriched (right) in SUM159 cells after a 30 min SHP099 treatment. Dark blue dots indicate phospho-peptides known to be involved in MAPK pathway regulation. (**F**) Immunoblot against phospho-p44/42 MAPK (Erk1/2)(Thr202/Tyr204) and ERK2 in SUM159 and MCF7 cells. Treatments were added 2 h prior to harvest (MEK162 1 μM, SHP099 2.5 μM) (*n* = 2). (**G**) A multi-protein signaling complex is assembled upon activation of receptor tyrosine kinases (RTKs), cytokine receptors, or scaffolding proteins. Adaptor proteins such as growth factor receptor-bound protein 2 (GRB2) and GRB2-associated-binding protein 1 or 2 (GAB1/2) engage with SHP2 and promote RAS activation via its guanine nucleotide exchange factor son of sevenless homolog 1 (SOS1). Activated Ras relays the signaling to the Raf/MEK/ERK signal transduction cascade. Upon phosphorylation, ERK can activate different transcription factors, one of them being ETS1. Phosphorylated ETS1 then initiates transcription of CXCL8, that is translated and secreted by cancer cells and may thus enhance metastatic progression of the disease.

## Declarations

D.B. is a full-time employee of Monte Rosa Therapeutics. R.J.A is a full-time employee of Novartis Pharma A.G. M.B.-A. owns equities in and has received laboratory support and compensation from Novartis, and serves as consultant for Basilea. M.B.-A. is an Editorial Board Member of the Journal of Mammary Gland Research and Neoplasia.

## Abbreviations

**BLBC**: Basal like breast cancers
**CXCL**: Chemokine (C-X-C motif) ligand
**CXCR**: Chemokine (C-X-C motif) receptor
**ETS1**: ETS proto-oncogene 1
**IL**: Interleukine
**ISMARA**: Integrated Systems for Motif Activity Response Analysis
**MAPK**: Mitogen-activated protein kinase
**MARA**: Motif activity response analysis
**MMP**: Matrix metalloproteinase
**RTK**: Receptor tyrosine kinase
**SHP2**: Src-homology 2 domain-containing phosphatase
**TNBC**: Triple-negative breast cancer
