# Supplementary figures and images for: Protein Tyrosine Phosphatase SHP2 Controls Interleukin-8 Expression in Breast Cancer Cells
Source: J Mammary Gland Biol Neoplasia. 2022 Jun 23;27(2):145–53. doi: 10.1007/s10911-022-09521-x (PMC9433352; doi:10.1007/s10911-022-09521-x)

## A

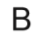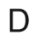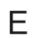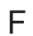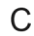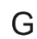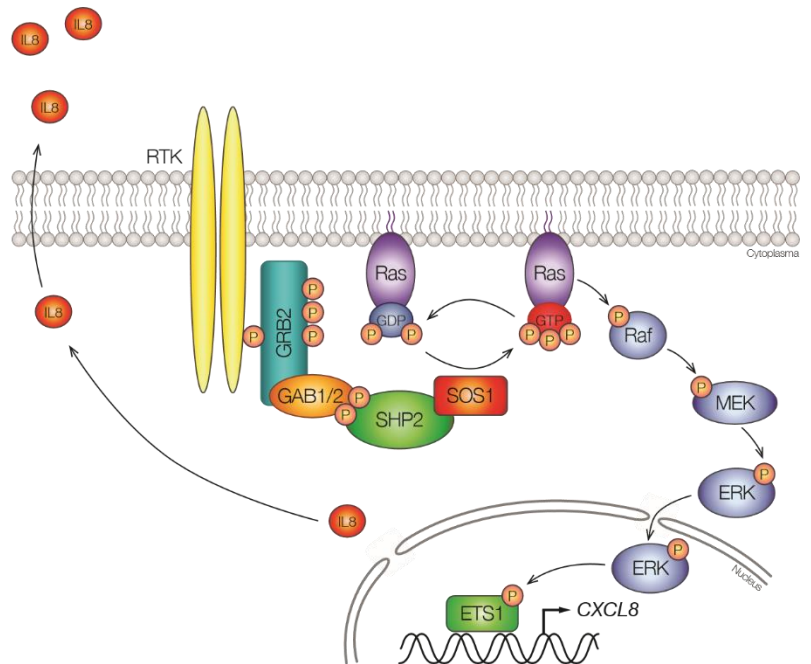

Supplement: Supplementary file 1 — SHP2 blockade downregulates MAPK and leads to reduced IL-8 levels in vitroand in vivo. (A) Bar graph representing PTPN11 (SHP2) mRNA expression in the indicated cell lines upon SHP2 knockdown. Data are shown ± S.D.(n = 3, ****P ≤ 0.0001, Two-way ANOVA test). (B) Differential expression of PTPN11 and CXCL8 upon SHP2 knockdown in SUM159 tumors and MCF10A-HER2/HER3 3D cultures. (C) Scatter plot representing ranked phospho-tyrosine peptides depleted (left) or enriched (right) in SUM159 cells upon SHP099 treatment at the indicated timepoints. Dark blue dots indicate phospho-peptides known to be involved in MAPK pathway regulation. The dotted line represents the used cutoff value of 1-fold change in log2 space. (D) Representative phospho-tyrosine peptides involved in MAPK pathway activation (orange) or inhibition (blue) upon SHP099 inhibition at the indicated time points. (E) Scatter plot representing phospho-serine/threonine peptides depleted (left) or enriched (right) in SUM159 cells after a 30 min SHP099 treatment. Dark blue dots indicate phospho-peptides known to be involved in MAPK pathway regulation. (F) Immunoblot against phospho-p44/42 MAPK (Erk1/2)(Thr202/Tyr204) and ERK2 in SUM159 and MCF7 cells. Treatments were added 2 h prior to harvest (MEK162 1 μM, SHP099 2.5 μM) (n = 2). (G) A multi-protein signaling complex is assembled upon activation of receptor tyrosine kinases (RTKs), cytokine receptors, or scaffolding proteins. Adaptor proteins such as growth factor receptor-bound protein 2 (GRB2) and GRB2-associated-binding protein 1 or 2 (GAB1/2) engage with SHP2 and promote RAS activation via its guanine nucleotide exchange factor son of sevenless homolog 1 (SOS1). Activated Ras relays the signaling to the Raf/MEK/ERK signal transduction cascade. Upon phosphorylation, ERK can activate different transcription factors, one of them being ETS1. Phosphorylated ETS1 then initiates transcription of CXCL8, that is translated and secreted by cancer cel [file 10911_2022_9521_MOESM1_ESM.pdf]
